# The Bitome: digitized genomic features reveal fundamental genome organization

**DOI:** 10.1093/nar/gkaa774

**Published:** 2020-09-25

**Authors:** Cameron R Lamoureux, Kumari Sonal Choudhary, Zachary A King, Troy E Sandberg, Ye Gao, Anand V Sastry, Patrick V Phaneuf, Donghui Choe, Byung-Kwan Cho, Bernhard O Palsson

**Affiliations:** Department of Bioengineering, University of California San Diego, La Jolla, CA 92093, USA; Department of Bioengineering, University of California San Diego, La Jolla, CA 92093, USA; Department of Bioengineering, University of California San Diego, La Jolla, CA 92093, USA; Department of Bioengineering, University of California San Diego, La Jolla, CA 92093, USA; Department of Bioengineering, University of California San Diego, La Jolla, CA 92093, USA; Department of Bioengineering, University of California San Diego, La Jolla, CA 92093, USA; Bioinformatics and Systems Biology Program, University of California San Diego, La Jolla, CA 92093, USA; Department of Biological Sciences and KI for the BioCentury, Korea Advanced Institute of Science and Technology, Daejeon 34141, Republic of Korea; Department of Biological Sciences and KI for the BioCentury, Korea Advanced Institute of Science and Technology, Daejeon 34141, Republic of Korea; Intelligent Synthetic Biology Center, Daejeon 34141, Republic of Korea; Department of Bioengineering, University of California San Diego, La Jolla, CA 92093, USA; Department of Pediatrics, University of California San Diego, La Jolla, CA 92093, USA; Novo Nordisk Foundation Center for Biosustainability, Technical University of Denmark, Lyngby 2800, Denmark

## Abstract

A genome contains the information underlying an organism's form and function. Yet, we lack formal framework to represent and study this information. Here, we introduce the Bitome, a matrix composed of binary digits (bits) representing the genomic positions of genomic features. We form a Bitome for the genome of *Escherichia coli* K-12 MG1655. We find that: (i) genomic features are encoded unevenly, both spatially and categorically; (ii) coding and intergenic features are recapitulated at high resolution; (iii) adaptive mutations are skewed towards genomic positions with fewer features; and (iv) the Bitome enhances prediction of adaptively mutated and essential genes. The Bitome is a formal representation of a genome and may be used to study its fundamental organizational properties.

## INTRODUCTION

A genome contains multiple classes of information that determine the form and function of an organism (1). Genome-scale experimental methods elucidate genomic features such as sequence (2), transcription units (3) and regulatory elements (4), among many others. This information is critical for genome-scale metabolic reconstructions (5,6), transcriptional regulatory network characterization (7) and genome design and reduction efforts (8,9), to name a few.

Currently, this genomic information centers around open reading frames and resides in text- and/or image-based formats, limiting comprehensive study of all genomic information. The genome is structured into macrodomains (10), and the location of a gene can affect its expression levels (11). For example, the Y-ome, defining thirty-four percent of *Escherichia coli* genes lacking functional evidence, is enriched near the terminal region (12). Fundamental genomic characteristics such as GC content are patterned periodically on different length scales (13). These findings motivate a formal, base-pair centered construct that represents features encoded by the entire genome sequence.

To address this need, we hereby introduce the Bitome, a matrix associating each genomic position in a sequence with the features it encodes. As an example, we constructed a Bitome for the *E. coli* K-12 MG1655 genome. We observed that: (i) genomic features are patterned unevenly across the sequence; (ii) the feature density of both coding and intergenic regions is revealed at base-pair resolution and differentiates sub-features in those regions; (iii) adaptive mutations occur more frequently at genomic locations encoding fewer total features; and (iv) the Bitome formalization allows prediction of adaptively mutated genes and gene essentiality based solely on sequence features. Thus, the Bitome is a novel construct that formally describes genomic feature information and lays the groundwork for actionable prediction based on that information.

## MATERIALS AND METHODS

### Assembling genome features

The *E. coli* strain K-12 substrain MG1655 reference genome (Reference Sequence NC_000913.3) was downloaded from NCBI in GenBank format. The reference was parsed using the *SeqIO.read* function from Biopython (14) (version 1.74). This reference genome defines the genomic positions. The following genomic features and their genomic locations were parsed from the reference genome: coding genes (CDS), pseudogenes, RNA-coding genes, insertion elements, repeat regions, and the origin of replication. Clusters of orthologous groups (COGs) functional annotations for genes from the reference genome were downloaded from NCBI (15) and linked via locus tag (b-number). Protein features were obtained from the GEM-PRO pipeline in the *ssbio* Python library (16) and linked to CDS from the reference genome by locus tag. Regulatory features were downloaded from RegulonDB (4) (version 10.0). The following regulatory features were parsed from RegulonDB: operons, transcription units, promoters (including −10 elements, −35 elements, and transcription start sites [TSS]), transcriptional and translational terminators, transcriptional and translational attenuators, Shine−Dalgarno sequences, riboswitches, transcription factor binding sites, and regulons (including sigmulons). Promoters not linked to a transcription unit were excluded. Genes from the reference genome were linked to operons and transcription units from RegulonDB via the locus tag. RegulonDB operons and transcription units not linked to a gene from the reference genome were excluded, and vice-versa. Independently-regulated gene modules (7) identified via independent component analysis (ICA) were linked to reference genome genes by locus tag.

### Constructing the Bitome

Genome features were assembled into a sparse matrix using SciPy's (17) sparse matrix package. Each row represents a different genomic feature, and each column corresponds to a genomic position. Each element *b*_*ij*_ in the matrix has a value of either 1 or 0; 1 indicates presence of feature *i* in column *j*, and 0 indicates absence To preserve the binary nature of the matrix (only 1s and 0s), features with multiple types were split into multiple rows as appropriate. For example, the 64 codons and 21 amino acids (this genome includes selenocysteine) were each represented in their own set of rows. To avoid overlaps and loss of information, certain features were split into six rows. These rows corresponded to three ‘frames’ (calculated as mod-3 of the start location) for each of the two strands (forward and reverse). Features treated in this manner were: genes, codons, proteins, amino acids (and all amino acid-based structural information), COGs. Regulatory features were represented in two rows corresponding to the forward and reverse strands. Regulons, sigmulons, *i*-modulons and transcription factor binding sites were left as single rows as no strand-specific information is available. Feature inclusion criteria and total counts of features included in the Bitome are listed in Supplementary Table S2.

### Computing sequence coverages

The ‘bit counts’ associated with each genomic position were calculated by taking the column-wise sum of the assembled matrix. Sequence coverages for selected features were computed by extracting a sub-matrix with just the rows corresponding to the features in question, summing the resulting sub-matrix row-wise, and computing the count of non-zero elements in the resulting vector along the length of the genome. Bit densities (in bits per bp) for genes and other genomic features were calculated by extracting a sub-matrix corresponding to the genomic range of the feature in question, computing the sum of that sub-matrix, and dividing by the length of the genomic range.

### Assembling and mapping ALE mutations

ALE mutations were downloaded from ALEdb (18) (version 1.0). SNPs based on reference sequence NC_000913.3 were selected. SNP density by genomic feature was calculated by determining the genomic positions with a 1 annotated for said feature (as described above) and dividing the total sequence length for that feature into the number of SNPs located at any of the feature's locations.

### Computing mRNA secondary structure

mRNA minimum free energy structures were calculated with Nupack (19) in sliding 100 bp windows across the reference genome. A genome-wide average G was calculated; ‘tight’ regions were defined as those with minimum free energies in the lowest 10%, genome-wide.

### Classifying genes with ALE SNPs

The scikit-learn (version 0.22.2) machine learning package was used to predict coding genes with ALE SNPs (20). For each of 4186 coding genes, the Bitome matrix region corresponding to that gene's location was extracted. Each gene matrix was summed column-wise to create a gene feature vector. These feature vectors were transposed and concatenated into a gene feature matrix with dimensions 4186 (coding genes) × 1634 (Bitome features). The gene feature matrix was min/max normalized. A target label vector was generated by checking the location range of each gene for a SNP in ALEdb; if at least one was found, a 1 was placed in the target label vector; 0 otherwise. There were 2923 coding genes observed with SNPs, and 1263 without. 20% of the data (evenly-weighted by class) was held out to generate a lockbox test dataset for final model evaluation.

The training data (gene feature matrix without lockbox data) still had a roughly 2-to-1 class imbalance. Thus, the majority class (SNP) was randomly down-sampled for all model training and cross-validation discussed below. Different classification models were evaluated for their performance on the training data. Adaptive boost, logistic regression, support vector machine, and random forest classifiers from scikit-learn - along with the XGBoost classifier from XGBoost version 1.0.2 (21) and an artificial neural network implemented with Tensorflow Keras - were run through 5-fold cross validation with five different downsampled training sets (Supplementary Figure S3A). This same cross validation was performed after shuffling target labels as a negative control to obtain the expected accuracy of 50% (guessing), and with only the nucleobase features included. Hyperparameters for all models were optimized using a 5-fold randomized search cross validation approach.

Final model performances were assessed by re-training each hyperoptimized model on five downsampled versions of the lockbox test set. Based on this assessment, a support vector machine with the following non-default parameters was selected as the final model: *penalty*=’l1’, *dual*=False, *C*=0.1. Model coefficients for assessing feature importance were accessed using the *coef_* attribute.

### Classifying essential genes

Essential gene labels were obtained from the Keio collection (22). The scikit-learn package was again used for the classification workflow. Train and test sets were defined the same way as for ALE SNPs, except that mean instead of sum was used to collapse each gene sub-matrix into a feature vector. There were 294 essential genes (class 1) and 3892 non-essential genes (class 0).

The same classifiers used for predicting ALE SNPs were tested for classifying essential genes. To address the large class imbalance, class frequency-weighted loss functions were used (for example, using the *class_weight*=’balanced’ argument for the scikit-learn classifiers). Models were initially assessed using 5-fold cross validation. Hyperparameters were optimized as with ALE SNPs.

Final performances were assessed by re-training each hyperoptimized model on the full training set and predicting based on the lockbox test set. Based on this assessment, a support vector machine with the following non-default parameters was selected as the final model: *penalty*=’l1’, *dual*=False, *C*=0.1, *class_weight*=’balanced’. Model coefficients for assessing feature importance were accessed using the *coef_* attribute.

## RESULTS

### The Bitome formalizes genomic features at the base-pair level

**Figure 1.**
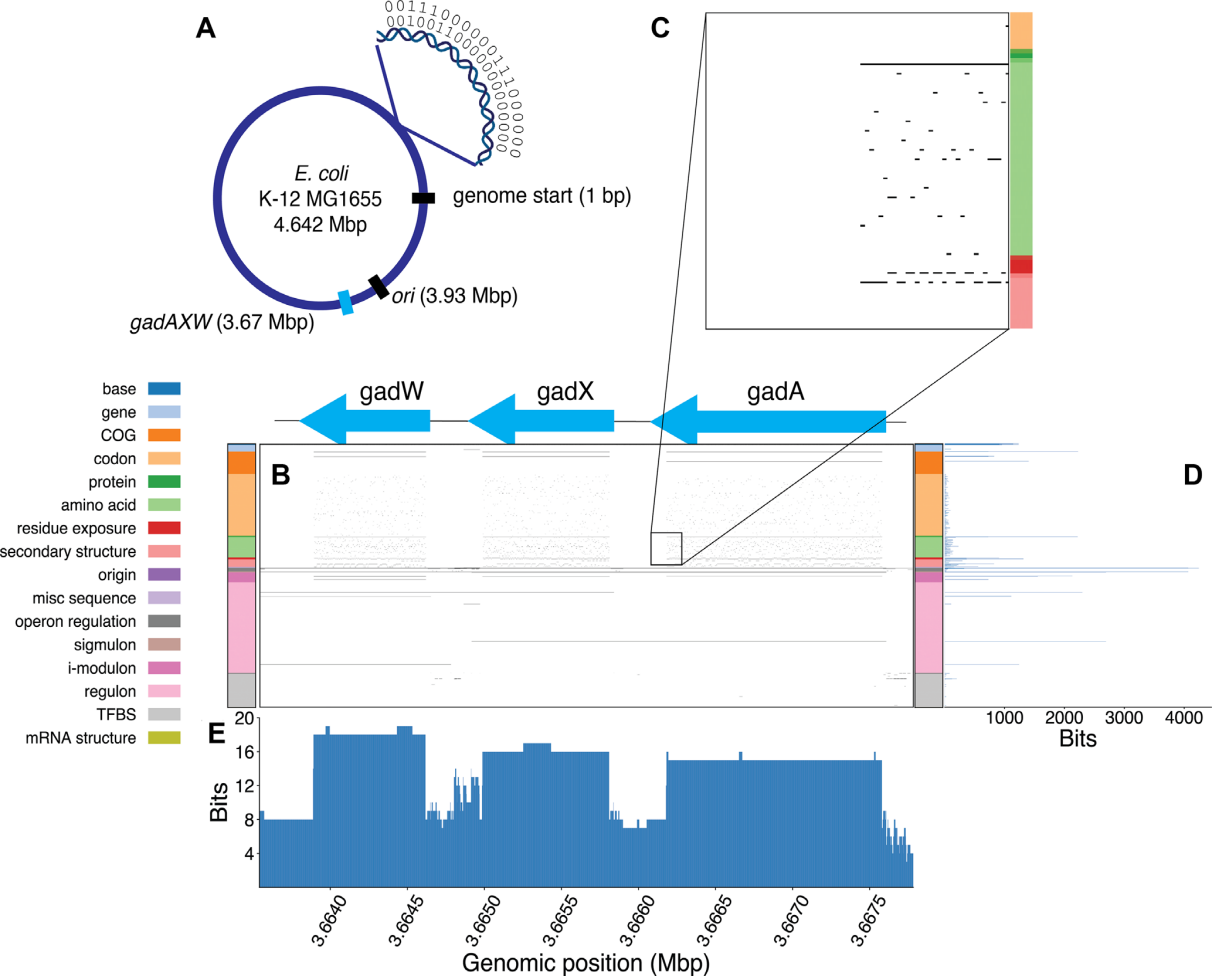
Features encoded by the *E. coli* K-12 MG1655 genome can be represented as a binary matrix. (**A**) *E. coli* K-12 MG1655 genome with reference genome start position, origin of replication (ori), and the *gadAXW* operon marked. (**B**) A visualization of the Bitome section at the location of the *gadAXW* operon. Rows are genomic features, columns genomic position. Black = 1, white = 0. (**C**) Close-up visualization of a 200 x 200 section of the Bitome section in (B). (D and E) Bit counts of the rows (**D**) and columns (**E**) of this section.

We constructed a Bitome for the *E. coli* K-12 MG1655 genome. Each row represents a different genomic feature, and each column a genomic position. Each element *b*_ij_ in the matrix has a value of either 1 or 0; 1 indicates presence of feature *i* in column *j*, 0 indicates absence (Figure 1A). As a result, we refer to the elements of the Bitome as ‘bits’. The genomic features represented are: (a) core sequence-derived features, such as codons; (b) experimentally-determined features, such as transcription factor binding sites and (c) computationally predicted features, such as protein secondary structure (Figure 1B). The K-12 Bitome has 1634 rows (genomic features, listed in Supplementary Table S1) and 4 641 652 columns (genomic positions), containing 52.4 million bits. It is sparse; only 0.7% of the bits have a value of 1.

### Genomic features are distributed unevenly

The *gadAXW* operon exemplifies the Bitome's structure. Row bit counts in this region vary widely, from the full 4243 (indicating presence of an operon) to 0 (e.g. binding sites for most transcription factors are absent) (Figure 1D). The coding regions have higher column bit counts than the intergenic regions (Figure 1E). Focusing on a Bitome region at the edge of a coding gene makes this difference clear (Figure 1C). The intergenic regions of this operon are relatively feature-rich. Multiple transcription factor binding sites and tight mRNA secondary structure are located within the *gadW*-*gadX* intergenic region. The maximum column bit count is significantly lower than the row dimension of the Bitome, indicating that in a particular genomic position, a small minority of the total genomic features contain bits.

**Figure 2.**
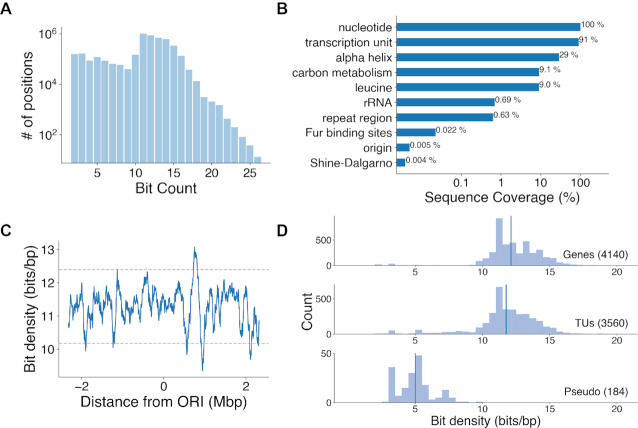
Bits are distributed unevenly. (**A**) Histogram of genomic positions by bit count. (**B**) Sequence coverage of 10 selected genomic features. (**C**) Moving average of bit density across the genome, calculated in 100 kb windows. Gray dashed lines indicate the mean ± 2 standard deviations. (**D**) Histograms of bit density for selected features (number of features indicated in parentheses). Vertical lines indicate medians.

In the entire Bitome, genomic position bit counts range from 2 to 26 (Figure 2A). Most genomic positions have between 10 and 15 bits. Far fewer genomic positions have >15. Significant variance is also present in the percentage of the total sequence that encodes different features. For example, genomic positions encoding carbon metabolism genes cover 9.1% of the genome, while Shine–Dalgarno sequences cover just 0.004% (Figure 2B). 35% of the genomic sequence codes for the hydrophobic amino acids leucine, alanine, glycine, valine and isoleucine (Supplementary Figure S1A). Alpha helices are confirmed as a common structural motif, encoded by 29% of genomic positions (Supplementary Figure S1B). The Bitome's organization also allows easy computation of sequence usages for overlapping features; for example, we observed that glycine is more common in loop regions than in either alpha helices or beta sheets (Supplementary Figure S1C). We performed hierarchical clustering of genomic positions within genes, transcription units, and operons; clusters were dominated by more dense features, such as amino acids, and did not conclusively associate distinct features.

### The bit density of coding and intergenic regions defines distinct sub-regions at high resolution

Bit density, measured in bits per base pair (bits/bp), varies across genome regions. For example, at 100-kb resolution, a moving average of bit density fluctuates (Figure 2C). The peak at 0.75 Mb is largely due to increased density of transcription units in that region (Supplementary Figure S2). Variation in bit density is not notably periodic. Bit density also differentiates between coding and intergenic features. Protein-coding genes and transcription units typically contain 12 bits/bp, while pseudogenes are less feature-rich (Figure 2D).

**Figure 3.**
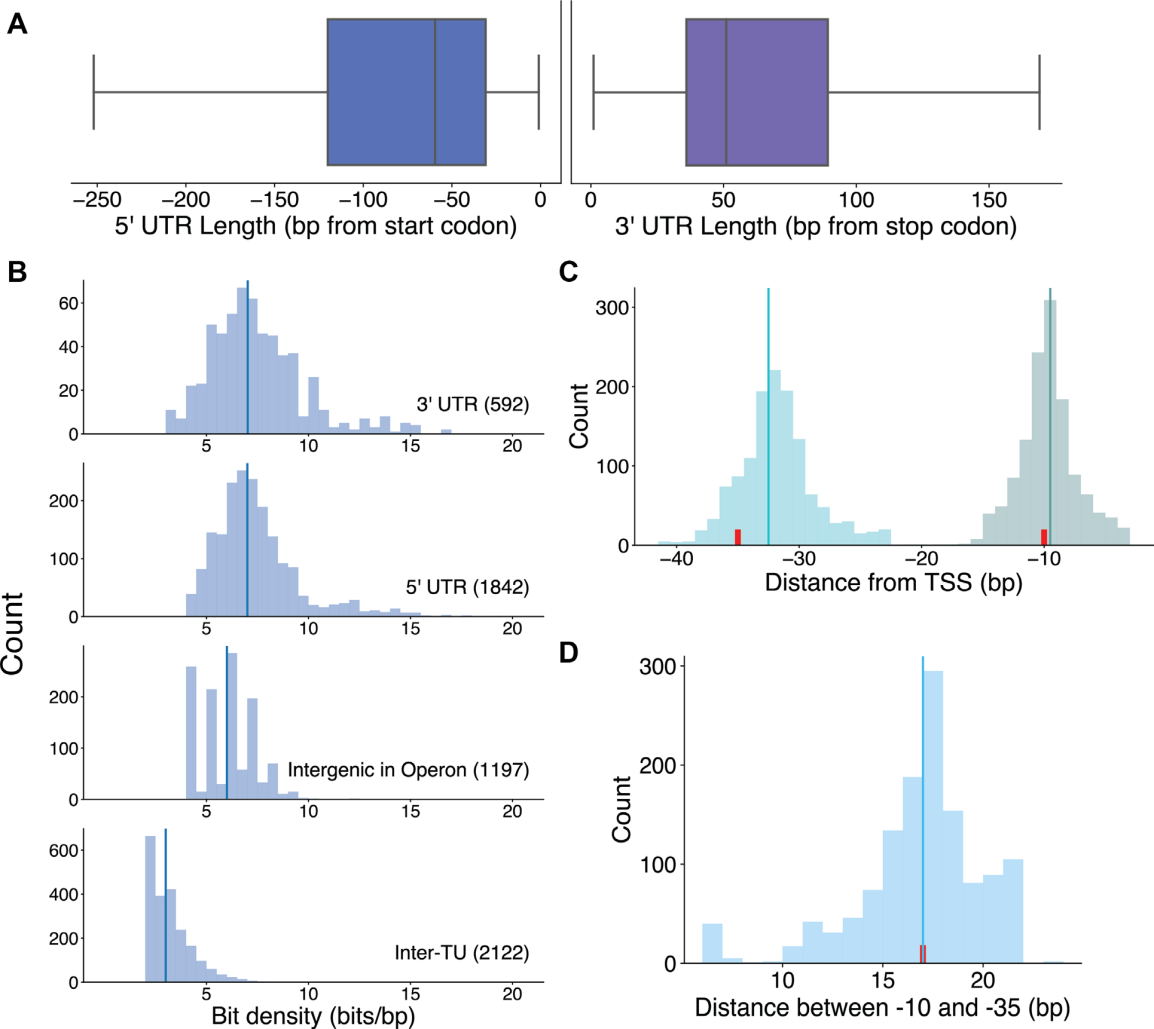
The Bitome provides a high-resolution view of bit density in intergenic regions. (**A**) Boxplots of the 5′ (blue) and 3′ (purple) UTR lengths. 5′UTR: *n* = 1842, 152 outliers excluded. 3′ UTR: *n* = 594, 94 outliers excluded. Outliers excluded based on 1.5*IQR from Q1 and Q3 (included range indicated by whiskers). (**B**) Histograms of bit density of selected intergenic regions (number of regions indicated in subplot titles). Vertical lines indicate medians. Bits from both strands are considered. (**C**) Histograms of the distributions of the −10 (light green) and −35 (cyan) elements of promoter regions. The center of the element is used to compute distance to TSS. Red ticks indicate the canonical locations of the elements, and vertical lines indicate medians. *n* = 1306. (**D**) Histogram of distances between −10 and −35 elements from the same promoter (as measured from ends of elements). Red tick indicates literature value. Vertical line indicates median. *n* = 1306.

The Bitome reveals the bit density in intergenic regions. For example, the 5′ and 3′ untranslated regions (UTR) flanking coding genes together define a transcription unit (TU) (3). These regions have median lengths of ∼50 bp and can be much longer (Figure 3A). Intergenic regions within TUs and the 5′ and 3′ UTRs contain ∼6–7 bits/bp (Figure 3B). Overall, including these UTRs and within-TU intergenic regions, TUs occupy ∼91% of the genome sequence (Figure 2B). Thus, 9% of the genome consists of ‘inter-TU’ regions.

The inter-TU regions are feature-deficient, having a median bit density of just 2.5 bits/bp (Figure 3B). Transcriptional regulatory sequences such as −10 and −35 elements reside in these areas. These sequences’ true locations differ slightly from their nomenclature, with the -35 elements especially tending to be found ∼2 bp closer to the transcription start site (TSS) (Figure 3C). The distance between these elements - shown to be important for RNA polymerase binding to the promoter region (23) - is an aspect of the inter-TU region recapitulated by the Bitome (Figure 3D). Despite the regulatory sequences, in most cases we only know the nucleotide sequence itself in inter-TU regions.

### Adaptive mutations occur at low-information genomic positions

**Figure 4.**
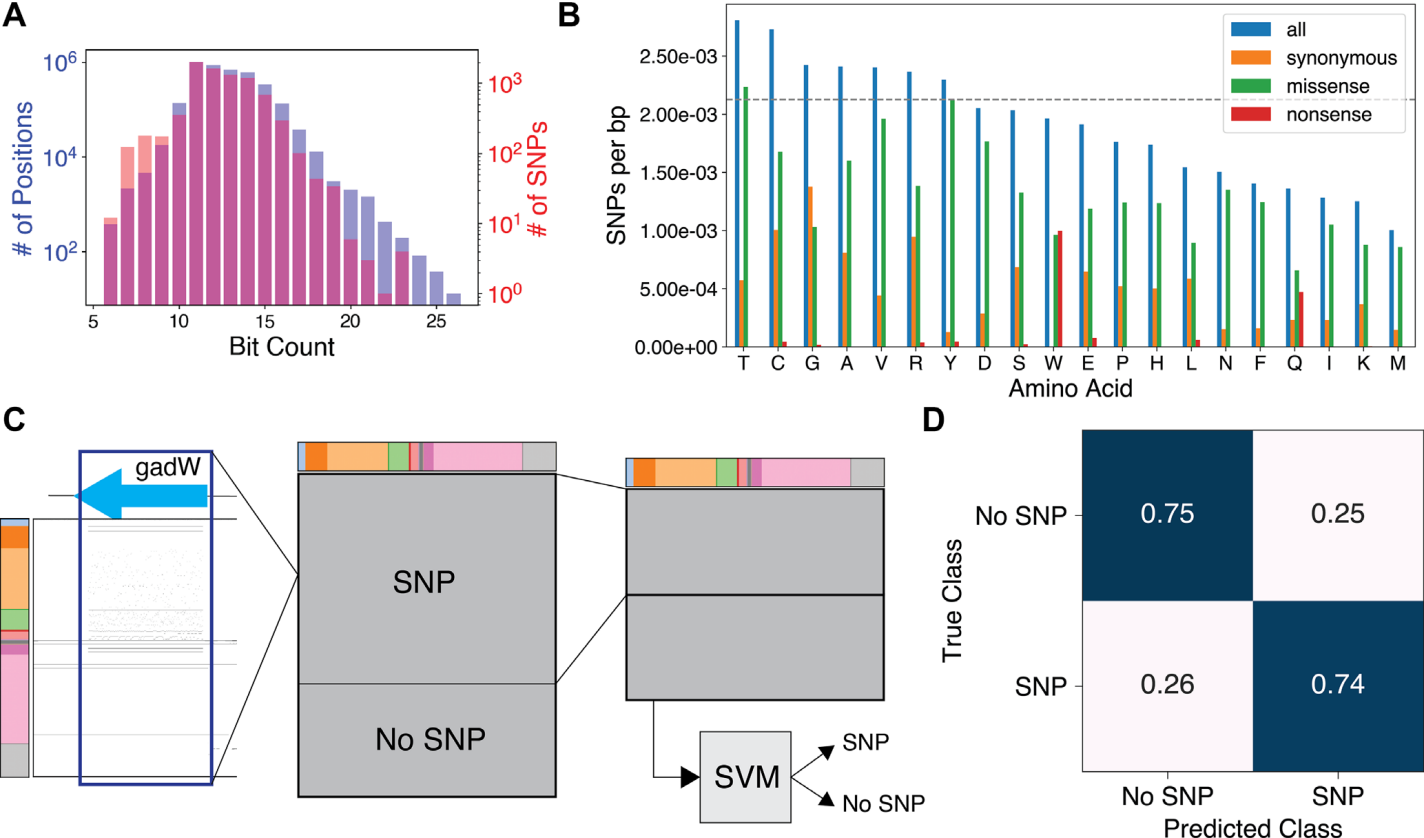
The Bitome enriches systemic analysis and prediction of adaptive mutations. (**A**) Combined histogram of the number of coding genome positions that contain the given number of bits (purple) and the numbers of SNPs that occur in coding positions with that number of bits (red). Two-sided Mann−Whitney *U* test: *P* = 0.015; *n* = 3 881 981, *m* = 7034. (**B**) Frequency of SNPs occurring at each amino acid. The gray dashed line is the overall frequency of SNPs across the entire genome. (**C**) Diagram of pipeline for predicting genes acquiring SNPs during ALE. From left to right: Bitome region for gene summed column-wise to give feature vector. Gene feature vectors combined into gene feature matrix and labeled as having at least one ALE SNP or not. Training matrix constructed by random down-sampling of majority class (SNP). Support vector machine (SVM) model trained to classify genes. Colorbar represents Bitome features as in Figure 1B. (**D**) Confusion matrix for final model. Scores are accuracy, normalized to true class. *n* = 506 in held-out, lockbox test set.

The Bitome sheds light on distal causation during adaptive laboratory evolution (ALE). Single nucleotide polymorphisms (SNPs) acquired during ALE experiments are distributed across the genome (18). Coding SNPs occur significantly less frequently at coding genomic positions with higher bit density (Figure 4A). Threonine, the sixth most abundant amino acid by sequence coverage, is the most frequently mutated amino acid, being mutated at a frequency higher than the overall sequence (Figure 4B). Conversely, leucine, despite being the most abundant amino acid by sequence coverage, is mutated at a frequency just two-thirds that of the genome as a whole. Hydrophobic residues are less frequently targeted by missense mutations despite presenting a larger sequence target.

### The Bitome aids prediction of adaptively-mutated and essential genes

The Bitome enables prediction of genes that acquire SNPs during ALE. Using just the bits from coding gene regions, we trained a support vector machine (SVM) classifier (Figure 4C) to distinguish between coding genes that do and do not acquire a SNP during ALE experiments. The SVM model performs this classification with 75% ± 1% accuracy (Supplementary Figure S3B) while not exhibiting a class bias (Figure 4D). The model maintains this accuracy when the nucleotides are removed; however, the model's performance worsens when just the sequence is used (Supplementary Figure S3A). Thus, the Bitome faithfully represents actionable genomic information coded by but not inferable from the sequence. Interestingly, the model identified the presence of the specific stop codon UAG as an important feature for predicting genes that have observed SNPs, while membership in the sigma factor 32 or Fis/Lrp/H-NS regulons is important for predicting non-mutated genes (Supplementary Figure S3C−E).

Similarly, essential genes identified in the Keio collection (22) were also classified with a support vector machine using the Bitome features. This classifier achieved an AUC of 0.75 (Supplementary Figure S4A), though it was less class-balanced than the SNP classifier, showing a bias toward the non-essential class (Supplementary Figure S4B). Nonetheless, the classifier identified reasonable clusters of orthologous groups (COGs) as important for prediction, such as cell cycle and translation (Supplementary Figure S4D). Interestingly, residue exposure also appeared as an important feature for classifying essentiality, highlighting the Bitome's potential to identify unexpected relationships between genomic features and phenotypic outcomes.

## DISCUSSION

In all, we find that the Bitome (i) reveals uneven encoding of genomic features and positions, (ii) recapitulates high-resolution feature density from both coding and intergenic regions, (iii) shows enrichment of adaptive mutations in feature-deficient genomic positions and (iv) facilitates prediction of adaptively mutated and essential genes. Similar to the stoichiometric matrix—used in genome-scale computational models to represent information about the reactome encoded in a genome (24)—the Bitome is a knowledge-type object that is binary and has no error. The stoichiometric matrix has been used extensively to characterize metabolic genotype-phenotype relationships through computation (25,26). As demonstrated above, the Bitome offers a similar approach to characterize the feature information encoded by a genome, while also enabling prediction from that information.

The Bitome is extensible in terms of the genomic features it represents, for it abstracts complex and varied features into a simple, coherent construct. More functional features could be included to identify more relationships between these and the core sequence-based features the Bitome currently contains. It is inherently applicable to other genomes to assess the distribution and nature of their features. Bitomes for other strains will allow for comparative analysis of feature content. Machine learning methods such as generative adversarial networks (27) could be trained on a series of Bitomes created for different strains to uncover principles of genome organization not observable in a single Bitome. Such principles could form a basis for design of novel genomes. Prediction of gene function across species - synteny - could also be improved by analyzing Bitomes at the gene cluster level. The Bitome is an organized and systematically represented form of genomic information and provides a platform to begin the process of deciphering ‘meaning’ from genomic sequences.

## DATA AVAILABILITY

New ChIP-exo data for transcription factor binding sites reported in this paper are deposited in the NCBI Gene Expression Omnibus with accession code GSE111095. Term-Seq data reported in this paper are deposited in the European Nucleotide Archive with accession number PRJEB36932. Code is available from the corresponding author upon request.

## Supplementary Material

gkaa774_Supplemental_Files
