# Supplementary material for: The Bitome: digitized genomic features reveal fundamental genome organization
Source: Nucleic Acids Res. 2020 Sep 25;48(18):10157–63. doi: 10.1093/nar/gkaa774 (PMC7544223; doi:10.1093/nar/gkaa774)
Supplement: gkaa774_Supplemental_Files [file gkaa774_supplemental_files.zip › Supplementary Data - Revised.pdf]

SUPPLEMENTARY DATA

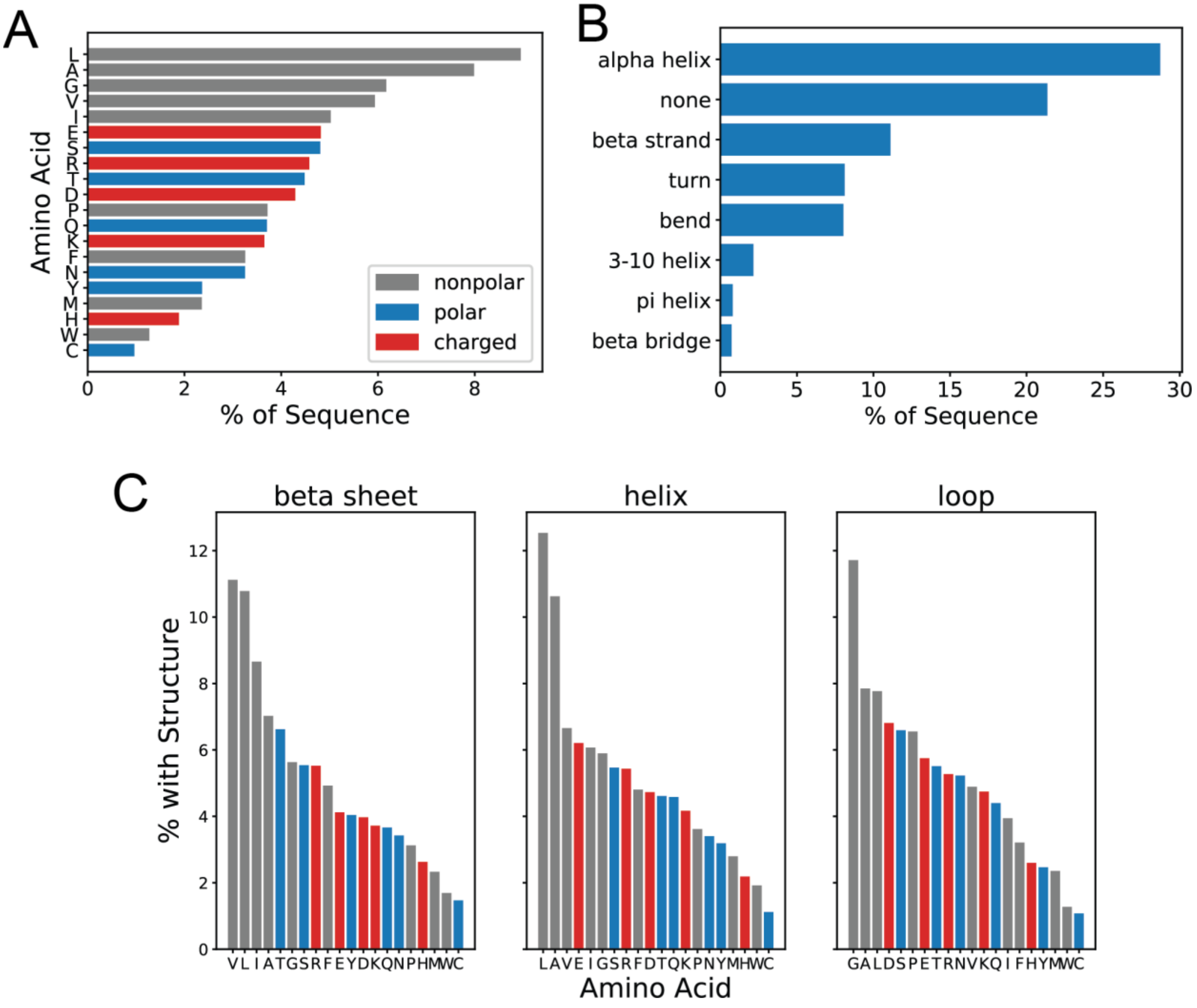

Figure S1. Amino acid and secondary structure features are easily cross-referenced. (A) Percentages of the total genomic positions coding for each of the amino acids. This calculation does *not* double-count genomic positions that code for the same amino acid on the forward and reverse strands. (B) Percentages of total genomic positions devoted to coding for different predicted secondary structures. Predictions from the DSSP algorithm (see *Methods*). (C) Amino acid preferences of secondary structural groups. See legend from A for color code. Beta sheet: beta bridge or beta strand; helix: alpha helix, 3-10 helix or pi helix; loop: turn or none.

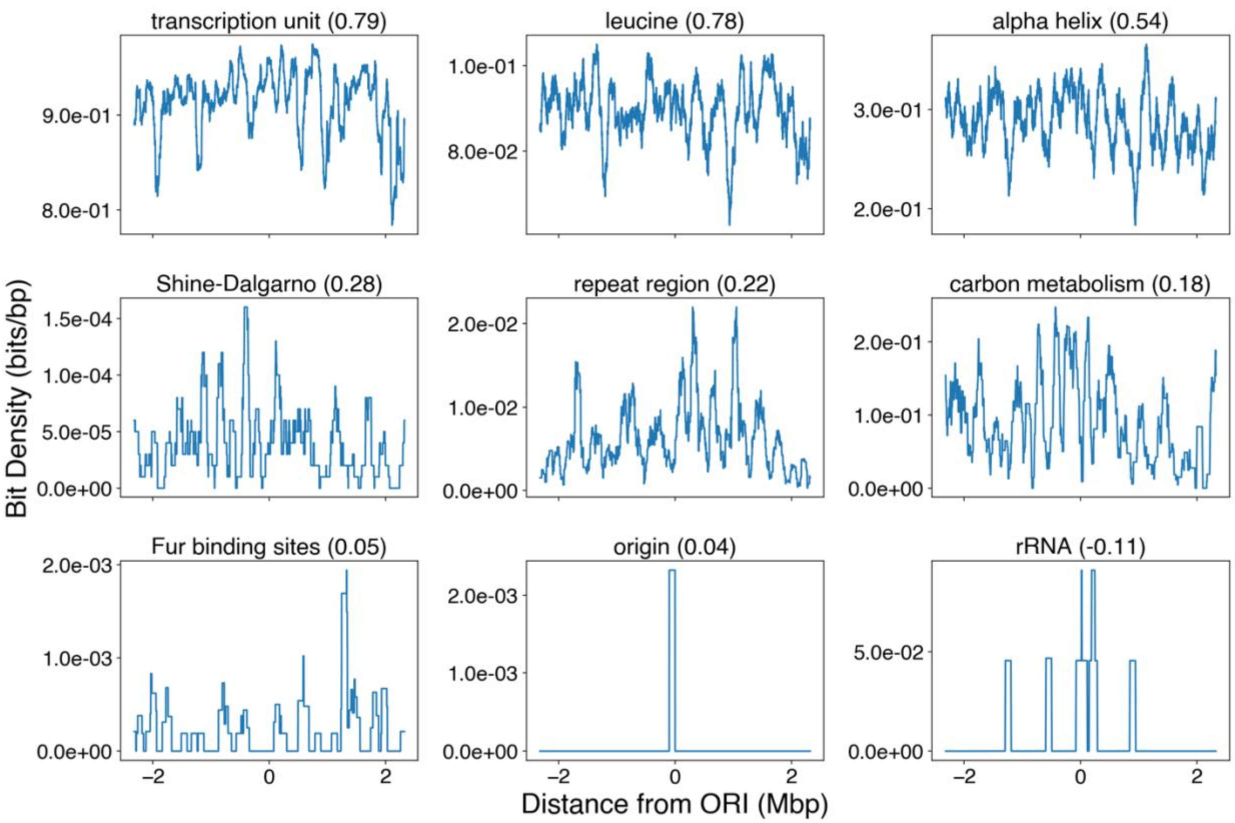

Figure S2. Selected genomic features are spatially organized differently. Moving averages in 100-kb windows of the selected features from Figure 2B. Value in parentheses is Pearson correlation coefficient between selected feature moving average and overall moving average (Figure 2C).

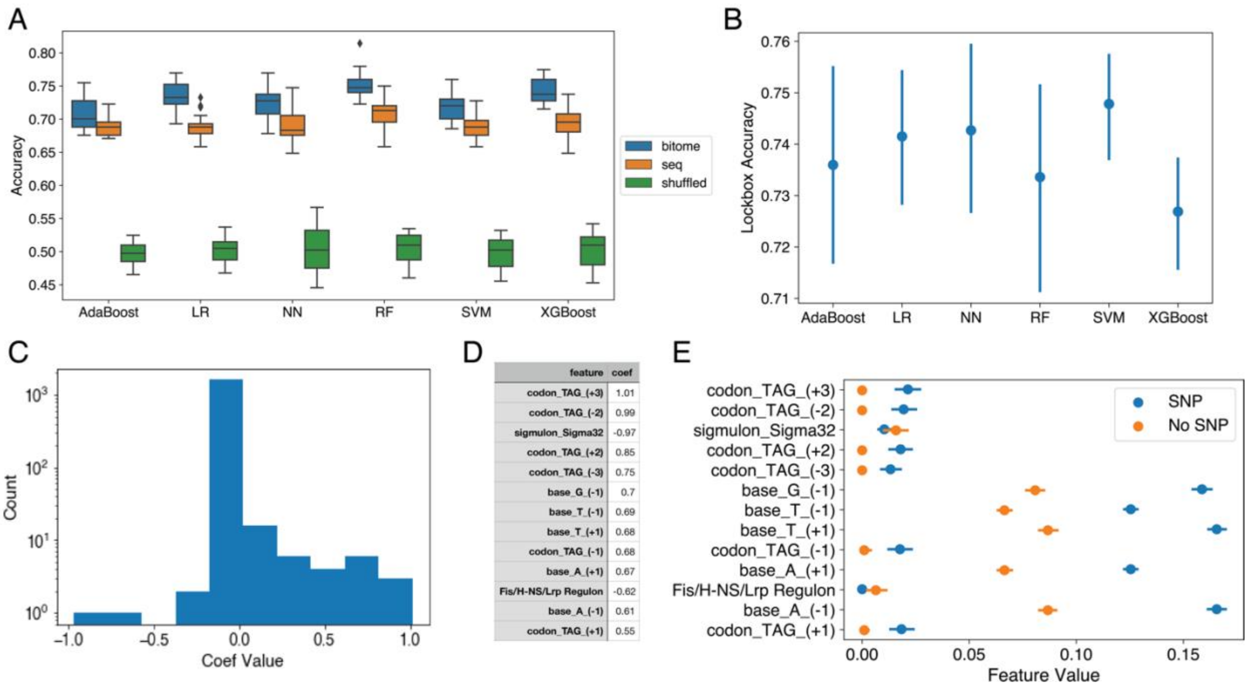

Figure S3. Support vector machine classifier selects Bitome features to predict genes with ALE SNPs. (A) Performances of 6 different out-of-the-box models at classifying genes with ALE SNPs. Bitome = used all bitome features; seq = sequence only; shuffled = shuffled target labels. n=25 for each group, composed of 5-fold cross validation for each of 5 downsamplings. AdaBoost = adaptive boosted tree; LR = logistic regression; NN = neural network; RF = random forest; SVM = support vector machine; XGBoost = extreme gradient-boosted trees. (B) Final performance of models on held-out, lockbox test data. Points are mean of performance on 5 downsamplings, bars are standard deviation. (C) Feature importances of final support vector machine classifier. (D) Table of Bitome features identified as important for classification. Positive coefficients indicate importance for predicting the SNP class, and negative coefficients indicate importance for predicting the No SNP class. (E) Class comparison of values (after min-max scaling) of the important features from panel D in the training set. Points are mean, bars are standard deviation. No SNP n=1010, SNP n=2339.

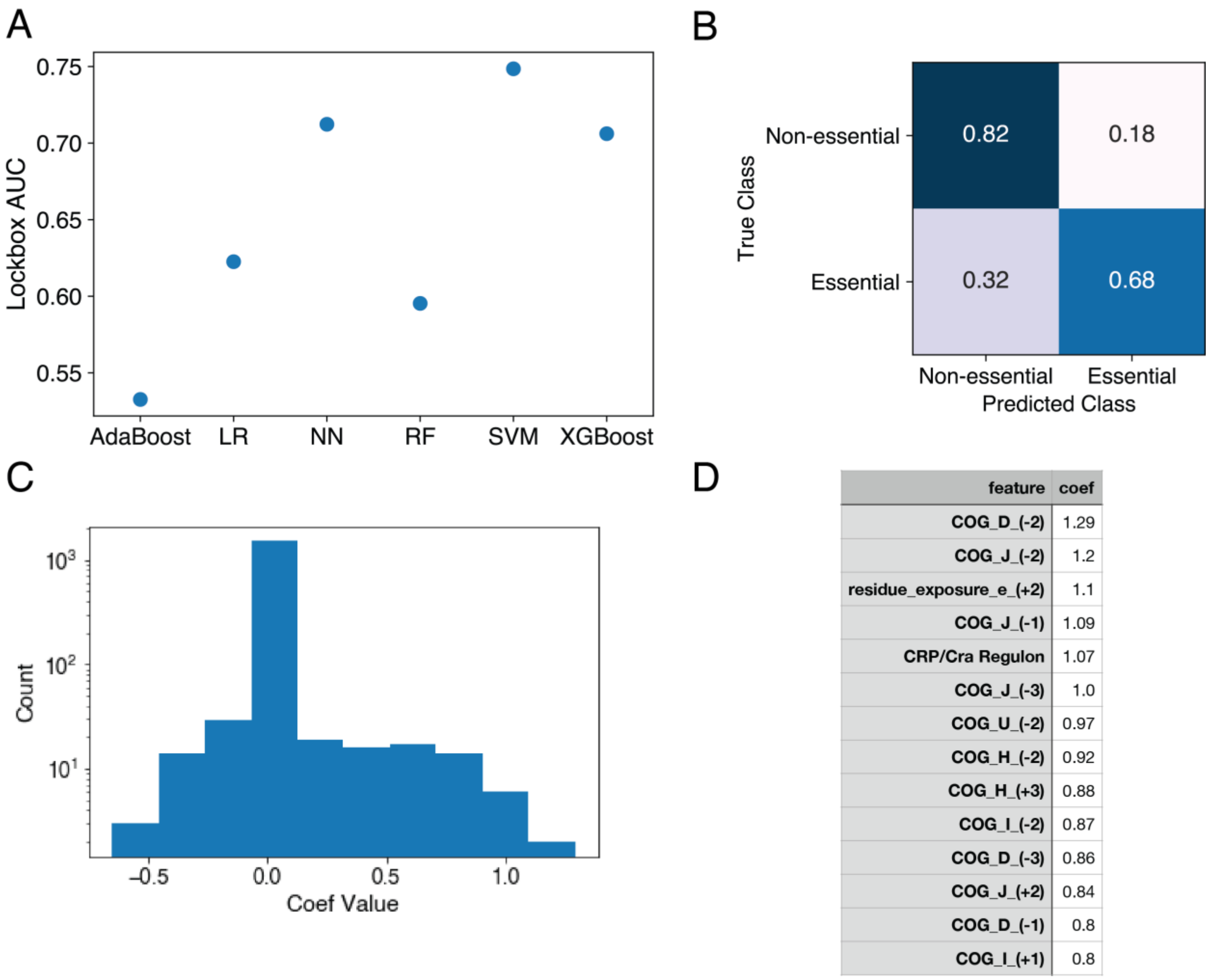

feature	coef
COG_D_(-2)	1.29
COG_J_(-2)	1.2
residue_exposure_e_(+2)	1.1
COG_J_(-1)	1.09
CRP/Cra Regulon	1.07
COG_J_(-3)	1.0
COG_U_(-2)	0.97
COG_H_(-2)	0.92
COG_H_(+3)	0.88
COG_I_(-2)	0.87
COG_D_(-3)	0.86
COG_J_(+2)	0.84
COG_D_(-1)	0.8
COG_I_(+1)	0.8

Figure S4: Classification of essential genes. (A) Final performances of models on held-out, lockbox test data. AUC = area under the receiver operating characteristic curve. (B) Confusion matrix for support vector machine classifier. Scores are accuracy, normalized to true class. n=838 in held-out, lockbox test set. (C) Feature importances of final support vector machine classifier. (D) Table of Bitome features identified as important for predicting essential genes. COG = cluster of orthologous groups; D = cell cycle control, cell division, chromosome partitioning; J = translation, ribosomal structure and biogenesis; U = intracellular trafficking, secretion, and vesicular transport; H = coenzyme transport and metabolism; I = lipid transport and metabolism.

Table S1. Included as separate file (Table S1.xlsx). Lists all genomic feature rows of the *E. coli* K-12 MG1655 Bitome in order of sequence coverage.

Table S2.

Feature	Sub-Feature	In Bitome	Total	Source	Inclusion Criteria
gene		4391	4496	GenBank	(for CDS) linked to >=1 RegulonDB TU protein
	COG	3497		JGI	
	i-modulon	1008		Sastry et al (2019)	
protein		4034	4139	GenBank	linked to a gene (see gene criteria)
	residue_exposure	3988		ssbio	
	secondary_structure	3921		ssbio	
origin		1	1	GenBank	all included
TU		3454	3560	RegulonDB	linked to included gene
operon		3454	2619	RegulonDB	linked to included TU
promoter		2053	8631	RegulonDB	linked to included TU
	-10_box	1351		RegulonDB	
	-35_box	1351		RegulonDB	
	sigmulon	1407		RegulonDB	
	regulon	1031		RegulonDB	
terminator		503	512	RegulonDB	linked to included TU
attenuator		1433	1466	RegulonDB	linked to included gene
Shine-Dalgarno		179	179	RegulonDB	linked to included gene
riboswitch		49	51	RegulonDB	linked to included gene
mobile_element		49	49	GenBank	all included
repeat_region		355	355	GenBank	all included
i-modulon		61	61	Sastry et al (2019)	all included
regulon		561	564	RegulonDB	
TF (active states)		209	406	RegulonDB/SBRG	>=1 annotated binding site
TFBS		3233	3233	RegulonDB/SBRG	all included
mRNA_structure		17429	17429	NUPACK	all included

Lists the total counts of genomic features taken from each data source, the criteria required to include each feature in the Bitome (if any), and the counts of features included in the Bitome representation based on said criteria.
